# Value of magnetic resonance angiography before prostatic artery embolization for intervention planning

**DOI:** 10.1038/s41598-024-58207-3

**Published:** 2024-04-02

**Authors:** Matthias Boschheidgen, Tim Ullrich, Rouvier Al-Monajjed, Farid Ziayee, Rene Michalski, Andrea Steuwe, Peter Minko, Peter Albers, Gerald Antoch, Lars Schimmöller

**Affiliations:** 1 Department of Diagnostic and Interventional Radiology, University Dusseldorf, Medical Faculty, Moorenstr. 5, 40225 Dusseldorf, Germany; 2 Department of Urology, University Dusseldorf, Medical Faculty, Moorenstr. 5, 40225 Dusseldorf, Germany; 3 Department of Diagnostic, Interventional Radiology and Nuclear Medicine, Marien Hospital Herne, University Hospital of the Ruhr-University Bochum, Herne, Germany

**Keywords:** Prostate MRI, Prostatic artery embolization, MR angiography, Benign prostatic hyperplasia, Prostate, Urinary tract, Urological manifestations

## Abstract

Knowledge about anatomical details seems to facilitate the procedure and planning of prostatic artery embolization (PAE) in patients with symptomatic benign prostatic hyperplasia (BPS). The aim of our study was the pre-interventional visualization of the prostatic artery (PA) with MRA and the correlation of iliac elongation and bifurcation angles with technical success of PAE and technical parameters. MRA data of patients with PAE were analysed retrospectively regarding PA visibility, PA type, vessel elongation, and defined angles were correlated with intervention time, fluoroscopy time, dose area product (DAP), cumulative air kerma (CAK), contrast media (CM) dose and technical success of embolization. T-test, ANOVA, Pearson correlation, and Kruskal–Wallis test was applied for statistical analysis. Between April 2018 and March 2021, a total of 78 patients were included. MRA identified the PA origin in 126 of 147 cases (accuracy 86%). Vessel elongation affected time for catheterization of right PA (p = 0.02), fluoroscopy time (p = 0.05), and CM dose (p = 0.02) significantly. Moderate correlation was observed for iliac bifurcation angles with DAP (r = 0.30 left; r = 0.34 right; p = 0.01) and CAK (r = 0.32 left; r = 0.36 right; p = 0.01) on both sides. Comparing the first half and second half of patients, median intervention time (125 vs. 105 min.) and number of iliac CBCT could be reduced (p < 0.001). We conclude that MRA could depict exact pelvic artery configuration, identify PA origin, and might obviate iliac CBCT. Vessel elongation of pelvic arteries increased intervention time and contrast media dose while the PA origin had no significant influence on intervention time and/or technical success.

## Introduction

Prostatic artery embolization (PAE) is an established therapeutic option for treatment of symptomatic benign prostatic hyperplasia (BPH). PAE is becoming an minimal invasive alternative to classical urological procedures such as transurethral resection of the prostate (TURP)^1–4^. Some studies reported a non-inferiority of the post-therapeutic results compared to surgical treatment in short-term follow-up while reducing the number of side effects^5–8^.

Nevertheless, PAE is a technically challenging procedure, and it remains unclear, which patients may be suitable for the intervention and benefit in the clinical long-term outcome. Exact knowledge about the vessel anatomy is supportive for interventional planning as vascular anatomy of iliac branches is heterogeneous and complex, and the origin of the prostatic artery varies between different PA types^9–11^. Furthermore, the interventional radiologist can prevent post-interventional complications by temporarily occluding collateral blood vessels to surrounding tissues (bladder, penis, and rectum) that become apparent during the intervention. It has been shown that an increase in iliac tortuosity measured in cone beam CT (CBCT) or pre-interventional CT angiography (CTA) leads to longer fluoroscopy times and radiation doses^12–15^. Kim et al. first proposed MR angiography before embolization to gather anatomic information about the prostatic vessels^16^. Zhang et al. examined the benefit of pre-interventional MRA and knowledge about prostatic vessel anatomy in a randomized trial when they showed that exact pre-interventional, MR-based information of the origin of the prostatic artery leads to lower radiation doses and shorter procedure times compared to a control group^17^. At our centre, patients standardly receive multiparametric magnetic resonance imaging (mpMRI) of the prostate before treatment to exclude presence of prostate cancer, so gadolinium enhanced MRA can be acquired in this setting and patients do not need an additional CT scan.

The aim of our study was to proof if pre-interventional MRA can identify the origin of the prostatic artery (PA) and if anatomic details i.e., iliac elongation, PA origin and bifurcation angles measured in MRA correlates with technical success of PAE and radiation dose and/or intervention time.

## Methods

### Study design

This retrospective single-centre study was approved by the local ethics committee (Faculty of Medicine, Heinrich-Heine University of Duesseldorf, Germany). All experiments were performed in accordance with relevant guidelines and regulations and the declaration of Helsinki. All patients provided informed consent prior to the study before image acquisition, allowing the scientific use of the acquired data. Between April 2018 and March 2021, patients who received prostatic artery embolization and prior MR angiography of the prostatic arteries before intervention were included in this study. All patients had severe symptoms of BPH, refractory to medical treatment, exhibited prostate volume ≥ 40 ml, and had been seen by an experienced urologist (*blinded*). Decisions for PAE were made in consensus between patients, urologists, and diagnostic/interventional radiologists. Patients were informed about the intervention at least 24 h before treatment and written informed consent was present from all patients. Prostate cancer was excluded prior to PAE by MRI and/or biopsy. Furthermore, asymptomatic patients, patients with acute prostatitis, renal insufficiency (GFR < 30 ml/min), neurogenic causes of BPS or insufficient coagulation status were excluded. All interventions were performed by an interventional radiologist with 5 years of experience in prostatic artery embolization (*blinded*).

Defined anatomic variables were measured retrospectively in pre-interventional MR scans. These variables included the Prostate Imaging Reporting and Data System (PIRADS) classification, prostate volume, image quality of MRA, PA origin, angles of aortic and iliac bifurcation, and vessel elongation. Outcome variables were extracted from data measured during DSA, which included PA origin, technical success, fluoroscopy time, number of cone beam CTs, dose area product (DAP), cumulative air kerma (CAK), total intervention time, intervention time for crossover and time for probing left and right PA separately and dose of applied contrast media.

The primary study aim was to correlate pelvic artery configuration (PA origin, iliac vessel elongation and angel) identified on pre-interventional MRA with the technical success and outcome variables (e.g., radiation dose or intervention time). Secondary objective was the determination of MRA accuracy for identification of the PA origin using DSA as gold standard.

### Imaging acquisition

All mpMRI scans were conducted on 3 T MRI scanners (Magnetom Prisma; Siemens Healthineers, Forchheim, Germany) using a 60-channel phased-array surface coil. MRI parameters were chosen according to international recommendations and contained T2-weighted turbo spin echo (TSE) sequences in 3 planes (T2WI), diffusion-weighted imaging (DWI)^18^ (Supp. Table 1). If cancer suspicion was present after acquisition of non-enhanced images, MRA with bolus tracking technique was acquired. A test bolus (2 ml CM, 2 ml/s injection rate; Clariscan; 0.5 mmol/mL; GE Healthcare) with subsequent single slice multiphase axial image acquisition (2D T1, TE 1.53 ms, TR 45.30 ms, FOV 350 mm, AT 40 s.) at the level of the infrarenal aorta above aortic bifurcation was acquired to determine the exact circulation time. Afterwards, a coronal angulated MRA (3D T1 FLASH, TE 1.24, TR 3.7 ms, slice thickness 0.9 mm, 112 slices, FOV 350 mm, 0.2 mmol/kg body weight CM dose plus 20 ml NaCl bolus, 2 ml/s injection rate) was conducted (approx. 20–30 s. after injection) after previous native acquisition. The field of view was placed over the lower abdominal aorta and iliac vessels and involved PA and entire prostate tissue (Supp. Fig. 1). Rotational maximum intensity projections (MIPs) were calculated for pelvic arteries.

### Prostate artery embolization

Prostatic artery embolization was conducted by the same interventional radiologist in all 78 patients. Technical aspects of the procedure have been described in detail previously^19^. All PAE were performed using an angiographic unit with a digital flat-panel detector system (Allura Xper FD20; Phillips Healthcare, Best, The Netherlands) equipped with cone beam CT option. First, the right common femoral artery (CFA) was punctured, and 5F-sheath was inserted in seldinger technique. Probing of left internal iliac artery was conducted using standardly a 5F-RIM and/or a 5F-SIM-1 with a hydrophilic guidewire. Next, DSA in an angulated series (LAO 30°, CRAN 10°) or CBCT (using 3D road map) was performed to identify the origin of the left PA with the catheter tip placed in the left internal iliac artery. Afterwards, a microcatheter (Direxion, Bern-Shape, 2.7/2.4 Fr) was coaxially inserted and probing of left the PA was performed using a microwire (Fathom 0.016’’). CBCT was executed applying 5 ml of diluted contrast (Imeron 400/NaCl; 50:50) at 0.2 ml/s to check embolization position and exclude collateral arteries. If collaterals were observed to penis, bladder or rectum, these branches were occluded temporarily using Gelfoam (Curaspon, CuraMedical B.V., Assendelft, The Netherlands)^20^. Microcatheter was placed distal in wedge position. Embolization was conducted using 250 µm-particles (Embozene Microspheres, Varian Medical Systems, Paolo Alto, CA) and subsequent 355–500 µm-Contour-particles (Boston Scientific, Natick, Massachusetts) until full stasis in the artery was achieved. Embolization was performed subsequently on the right side in the same way. In case of difficult probing of the prostatic arteria alternative microcatheters (Direxion, Swan-shape, 2.7/2.4 Fr or Echelon, 45° Tip, 1.7/2.1 Fr) and guidewires (Synchro soft 0.014″; Synchro 0.010″) were used. In case of insufficient probing/catheter positioning (e.g., due to stenosis) or if protective embolization of collateral arteries was unfeasible on one or both sides, prostate embolization was not conducted, respectively. After completing embolization all extraneous material was eliminated, and the puncture side was closed using 6F-Angioseal (Terumo, Somerset, New Jersey, USA).

### Analysis of MRI/MR angiography

**Figure 1:**
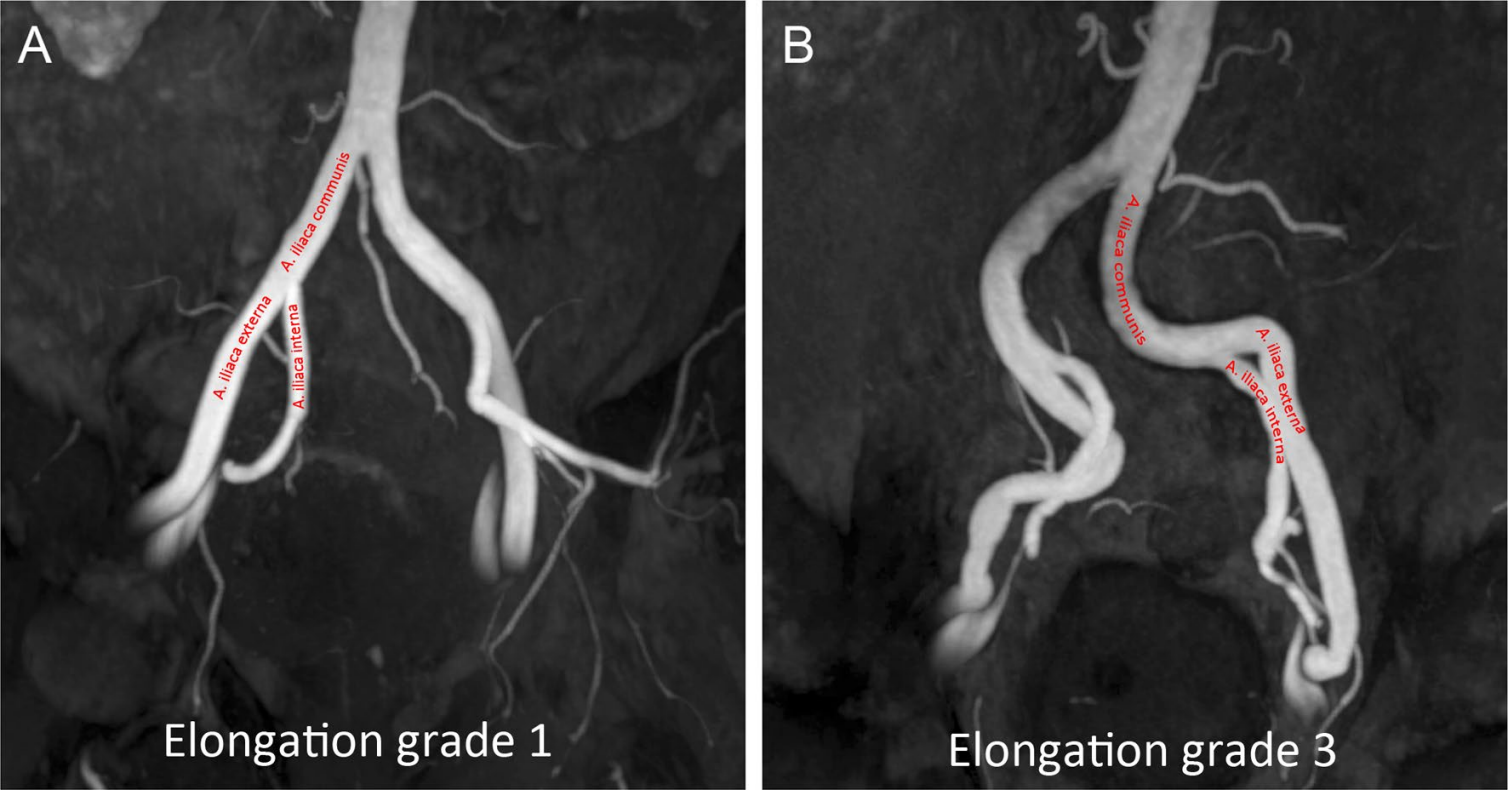
Different grades of iliac vessel elongation and kinking (1 = mild, 2 = moderate, 3 = severe) with examples of elongation grade 1 (**A**) and 3 (**B**).

**Figure 2:**
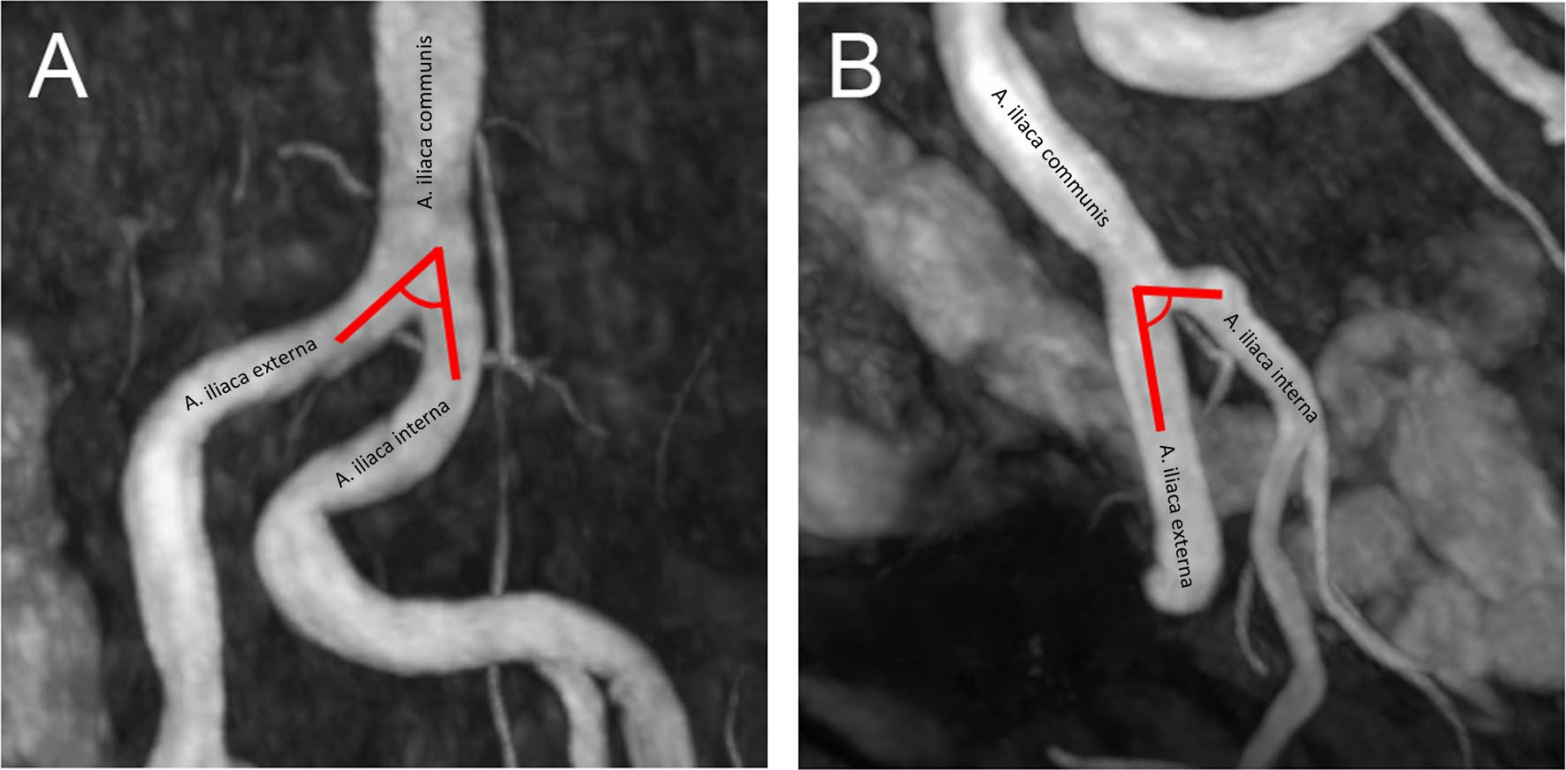
Measurement of iliac bifurcation angles: examples with a sharp (**A**) and less sharp (**B**) angle of the internal iliac artery.

PI-RADS classification and the prostate volume have been prospectively assessed. MR angiography was analysed retrospectively by three readers (*blinded*) with 3, 8, and 10 years of experience in mpMRI of the prostate, one of whom was the interventionalist. First, image quality of MRA was rated on a three 3-point scale, dividing into good, moderate, and insufficient^21^. This was conducted taking into consideration the severity of artefacts and the quality of contrast due to technical adjustment during acquisition. In case where image quality was inappropriate, the data was excluded from further analysis. Second, the origin of prostatic artery (PA) was defined according to Bilhim et al. and Carnevale et al. (PA type 1–4)^9,22^. Decisions were made in consensus in case of discrepant readings, which was the case in 12 patients. If more than one PA was present on one side, only the main (larger) PA was defined as the type of origin. If the PA origin fits no classic type, it was documented as “other”. Third, subjective iliac vessel elongation was ranked on a 3-point grading system dividing into mild, moderate, and severe elongation (grade 1–3) (Fig. 1). The readers performed 3D reconstruction of pelvic vessels to adequately measure angles of aortic bifurcation and iliac bifurcation on both sides. Measurements were performed placing a centreline in both vessels with the apex of the angle at the point of intersection of both angles (Fig. 2).

### Analysis of cone beam CT/digital subtraction angiography

CBCT and DSA images were evaluated by the same three radiologists (*blinded*), subsequently after MRA analysis. The PA was identified, and its origin was taken as reference. In case of discrepant reading, decision was made in consensus, which was necessary in 8 patients. PAE was defined as successful when embolization was executed on the considered side. Analysis was done independently for both sides. CAK, DAP, fluoroscopy time, dose of contrast media and total intervention time was measured for the entire procedure. For subanalysis of both sides autonomously, intervention time for crossover, and intervention time for probing of PA was quantified separately. Probing of PA included the time from probing common iliac artery until the catheter tip was placed in the PA. As we observed a reduction in the number of cone beam CT performed from the internal iliac artery, we decided to conduct an additional retrospective subgroup analysis comparing radiation doses in the first half of the patients with the second half. Therefore, we divided our collective into two equal groups. Allocation was conducted in chronological order. The first half included interventions performed between April 2018 and November 2019, the second half between December 2019 and March 2021.

### Statistical analysis

Statistics were performed using IBM SPSS^®^ Statistics (Version 27 IBM Deutschland GmbH). Data were checked for normal distribution. Descriptive statistics included mean, median, standard deviation and interquartile ranges. p-values < 0.05 were defined as statistically significant. Kruskal–Wallis test was performed to compare outcome parameters for different PA origin and elongation grades. For correlation analyses, the Pearson correlation coefficient τ was calculated. Correlation strengths r were graded as suggested by Cohen: small (r < 0.3), moderate (r = 0.3–0.5), and large (r > 0.5)^23^.

## Results

### Patients

Baseline characteristics of all seventy-eight patients are shown in Supplemental Table 2. PIRADS classification of 3 was present in 3 patients and PIRADS classification of 4 was present in 1 patient. These patients received biopsy prior to embolization to exclude prostate cancer. In the rest of the cohort, mpMRI revealed PIRADS classification of 1 or 2. Prostate volume differed widely in this collective, the smallest prostate measured 45 ml while the largest prostate had a volume of 293 ml.

### Evaluation of MR-angiography

The PA origin was divided into four different types according to Bilhim et al. and Carnevale et al.^9,22^. Overall image quality of MRA rated on a subjective 3-point-scale was good in 52/78 patients. In one patient, image quality of MRA was inadequate leading to the exclusion of the patient. PA was identified in MRA in 74/77 patients on the right side and 75/77 patients on the left side. In the rest of the cases, no PA origin could be determined. Compared to DSA as the reference standard, MRA could identify 126 of 147 PA origins (accuracy 86%; 95%-CI 79–91%). Origin of PA could not be identified in DSA in two cases either. Distribution of PA origin revealed a wide spread between the different PA types (Table 1). Median angle of aortic bifurcation was measured with 36 degrees (IQR 27–44 degrees), median angles of iliac bifurcation were 38 degrees on the right side (IQR 28–45 degrees), and 36 degrees on the left side (IQR 30–47 degrees), respectively. Cross table for anatomic PA types and differences between MRA and DSA is displayed in the supplemental data (Supp. Tables 3 and 4).

**Table 1:** Analysis of image quality and vessel characteristics on MRA.

IQ	Grade 1–3 median (IQR)	1 (1–1)
PA	Visibility n (%)	
	Right	74/77 (96)
	Left	75/77 (97)
	Type 1 n (%)	
	Right	23 (30)
	Left	20 (26)
	Type 2 n (%)	
	Right	24 (31)
	Left	17 (22)
	Type 3 n (%)	
	Right	14 (18)
	Left	20 (26)
	Type 4 n (%)	
	Right	13 (17)
	Left	17 (22)
	Other n (%)	
	Right	0 (0)
	Left	1 (1)
Angles	Aortic bifurcation, degree Median (IQR)	36 (27–44)
	Interna iliacal artery, degree Median (IQR)	
	Right	38 (28–45)
	Left	36 (30–47)
Elongation	Grade 1–3 Median (IQR)	2 (1–3)

MRA = magnetic resonance angiography; IQ = image quality; PA = prostatic artery; IQR = interquartile range.

### Technical data of PAE

PA origin determined in CBCT/DSA can be seen in Table 2. PA was identified in 77/ 78 patients on the right side and 76/78 patients on the left side. Successful embolization was performed on both sides in 81% of the patients (n = 63), only on the left side in 5 patients and only on the right side in 7 patients. In 5% (n = 4) of the patients, probing of PA was impossible on both sides (n = 4). For identifying and catheterization of PA and exclusion of relevant collateral vessels to other organs, CBCT was required with a median number of 4 per patient. Technical difficulty varied substantially between the different patients. This is reflected in a widespread of radiation dose and fluoroscopy time. Median DAP was 12,190 cGycm^2^ ranges from 5740 cGycm^2^ to 28,256 cGycm^2^ (IQR 9194–15,044 cGycm^2^). Fluoroscopy time was in median 33.9 min (IQR 27.3–46.5 min) and intervention duration was 115 min (IQR 90–135 min) (Table 2). The first half of included interventions performed between April 2018 and November 2019 had a median intervention time of 125 min (IQR 99–146 min) while the second half between December 2019 and March 2021 had a median intervention time of 105 min (IQR 90–125 min) (p < 0.001). The mean number of CBCT performed per patients could be reduced as well in the second period (p < 0.001), although the median number of CT conducted remained unchanged (n = 4). Mean duration of crossover manoeuvre was 10 min; mean probing time for left PA was 25 min, for right PA 21 min, accordingly. There was no significant difference between both sides. PA origin was identically for both sides in 57%; in the rest of the patients PA anatomy differed in its origin (Supp. Table 4).

**Table 2:** Analysis of vessel characteristics, embolization success, and technical parameters of DSA.

PA	Visibility n (%)	Right	77/78
		Left	76/78
	Type 1 n (%)	Right	28 (36)
		Left	24 (31)
	Type 2 n (%)	Right	23 (29)
		Left	14 (18)
	Type 3 n (%)	Right	14 (18)
		Left	18 (23)
	Type 4 n (%)	Right	12 (16)
		Left	18 (23)
	Other n (%)	Right	0 (0)
		Left	2 (3)
Successful embolization n (%)		Both	63 (81)
		Only right PA	5 (6)
		Only left PA	7 (9)
CBCT; number Median (IQR)		4 (4–4)	
DAP; cGycm^2^ Median (IQR)		12,190 (9194–15,044)	
CAK; mGy Median (IQR)		989 (703–1291)	
Fluoroscopy time (min) median (IQR)		33.9 (27.3–46.5)	
Intervention time; min Median (IQR)	Total	115 (90–135)	
	Crossover manoeuvre	7 (5–13)	
	Left	23 (17–30)	
	Right	18 (13–25.5)	
CM dose; ml Median (IQR)		75 (63–95)	

DSA = digital subtraction angiography; PA = prostatic artery; CBCT = cone beam CT; DAP = dose area product; CAK = cumulative air kerma; CM = contrast media; IQR = interquartile ratio.

### Evaluation of PA origin, iliac vessel elongation, and angles

For final analysis, data from 77 patients were evaluated. Descriptive data for technical outcome parameters divided for different PA origins and elongation grades is shown in Table 3. Kruskall-Wallis-test revealed no significant differences in technical parameters as radiation dose or applied contrast media between different PA origins. When considering absolute values, PA type 4 from internal pudendal artery tends to have shorter intervention times in general with a mean of 108.6 min, although these differences failed to reach statistical significance (p = 0.10). When focussing on the subjective severity of pelvic vessel elongation, fluoroscopy time (p = 0.05), volume of applied contrast agent (p = 0.02) and probing time of right PA (p = 0.02) increased significantly with the elongation grade. Furthermore, there was a trend towards higher overall intervention time (p = 0.10) with increasing elongation grade. Flat left and right iliac bifurcation angle showed a moderate and significant correlation for both DAP and CAK, implying a more challenging probing of PA in these patients, leading to an increase of radiation dose (DAP: r = 0.30 left with p = 0.02; r = 0.34 right with p = 0.01; CAK: r = 0.32 left; r = 0.36 right, both sides p = 0.01) (Table 4).

**Table 3:** Technical parameter of DSA for different PA types and elongation grades.

	Type of PA origin/grade of elongation	Fluoroscopy time (min) Median (IQR)	Intervention time (min) Median (IQR)	Intervention time crossover (min) Median (IQR)	Intervention time left PA (min) Median (IQR)	Intervention time right PA (min) Median (IQR)	DAP (cGycm^2^) Median (IQR)	CAK (mGy) Median (IQR)	CM dose Median (IQR)
PA	1 (n = 44)	36.2 ± 11.7	119.6 ± 23.1	9.5 ± 9.9	23.8 ± 6.7	22.8 ± 14.1	14,002 ± 6436	1193 ± 756	79.7 ± 23.2
	2 (n = 42)	36.5 ± 17.2	116.6 ± 35.8	11.2 ± 9.5	24.0 ± 13.4	22.8 ± 10.1	10,974 ± 2643	840 ± 218	79.7 ± 23.2
	3 (n = 35)	34.4 ± 15.3	115.0 ± 32.0	9.8 ± 0.5	22.2 ± 9.2	23 ± 13.9	14,164 ± 6044	1256 ± 914	89.5 ± 15.4
	4 (n = 30)	34.3 ± 8.3	108.6 ± 25.5	10.9 ± 7.8	20.7 ± 6.9	20.3 ± 7.6	13,401 ± 5739	778 ± 383	80.8 ± 12.5
Elongation	1 (n = 21)	28.8 ± 10.6	110.3 ± 32.3	7.0 ± 5.6	22.3 ± 19.9	16.3 ± 5.5	13,110 ± 4066	999 ± 424	69.4 ± 16.1
	2 (n = 34)	33.8 ± 12.2	111.3 ± 28.3	10.1 ± 8.4	22.1 ± 10.9	19.9 ± 10.4	12,257 ± 4902	966 ± 503	82.2 ± 20.7
	3 (n = 32)	45.0 ± 14.8	122.1 ± 24.4	13.8 ± 11.2	28.6 ± 11.7	21.8 ± 15.1	13,268 ± 4917	1175 ± 811	84.9 ± 14.8

DSA = digital subtraction angiography; PA = prostatic artery; DAP = dose area product; CAK = cumulative air kerma; CM = contrast media; IQR = interquartile range.

**Table 4:** Kruskal–Wallis test and correlation between MRA and DSA.

p-values	Interven-tion time left PA	Interven-tion time right PA	Interven-tion time total	Time cross-over	Fluro-scopy time	DAP	CAK	CM dose
PA type 1–4 per site	0.77	0.98	0.90	0.89	0.94	0.82	0.35	0.61
Elongation	0.16	**0.02**	0.10	0.62	**0.05**	0.52	0.58	**0.02**
Angle								
Aortic	0.09 r = − 0.22	0.52 r = − 0.09	0.44 r = − 0.11	0.12 r = − 0.21	0.33 r = − 0.13	0.88 r = − 0.02	0.89 r = 0.02	0.28 r = 0.03
Iliacal left	0.78 r = 0.04	0.27 r = 0.15	0.17 r = 0.18	0.44 r = 0.10	0.08 r = 0.23	**0.02** r = 0.30	**0.01** r = 0.32	0.98 r = − 0.00
Iliacal right	0.24 r = 0.16	0.37 r = 0.12	0.89 r = − 0.02	0.48 r = − 0.10	0.16 r = 0.18	**0.01** r = 0.34	**0.01** r = 0.36	0.25 r = − 0.15

Significant values are in bold.

Presented values are p-values; Pearson-correlation was used to check for the relation between angles and DSA-parameters; Kruskall-Wallis test was used to heck for the relation between PA type and Elongation with DSA parameters.

MRA = magnetic resonance angiography; DSA = digital subtraction angiography; PA = prostatic artery; DAP = dose area product; CAK = cumulative air kerma; CM = contrast media.

## Discussion

We evaluated pre-interventional pelvic MR angiography to identify the origin of prostatic artery and to determine pelvic artery configuration. MRA can provide important information about technical difficulty, success of the embolization and might reduce radiation dose by obviating iliac CBCT.

Consistent with other publications, we could show that MRA is able to classify the PA origin in 86% of the included patients^16,17^. Zhang et al. revealed that MRA was able to provide important information about pelvic vessel anatomy and this knowledge contributed reducing radiation doses and intervention times significantly. Main advantages of MR angiography compared to pre-interventional CT angiography (CTA) are the following: first, mpMRI of the prostate is being conducted in most patients to exclude significant prostate cancer before performing PAE^24,25^. Hence, an additional scan at another modality for visualizing prostatic arteries is not necessary. Second, computed tomography also implies another exposure to ionizing radiation. This can be avoided by using MRA. Regardless of the utilized modality, pre-interventional information of pelvic anatomy is important and may facilitate PAE planning to reduce radiation dose and intervention time. This saves important resources and helps to prevent patients from unnecessary radiation exposure^21,26,27^.

Patients with increased elongation of iliac vessels rated on a subjective 3-point scale faced higher radiation dose and procedure times. This stands in line with previous studies who investigated atheroma severity and vessel tortuosity in pre-interventional CTA and revealed effects on technical parameters and technical success of the intervention^12–14,21^. To our knowledge, this is the first study which examined this coherence with MR angiography. This means, that MRA is able to identify good candidates for PAE and predicts cases, where catheterization might be challenging and probably less promising. However, vessel elongation is no reason to exclude patients from embolization, as there was no difference in technical success and clinical benefit was not part of this study.

Looking on the bifurcation angles, Pearson correlation revealed moderate correlation between angles of iliac bifurcation on both sides and CAK and DAP. A possible explanation could be the increase in difficulty of probing flat angles. In these cases, a higher number of DSA series was necessary to identify the right artery, leading to an increase in radiation dose, but not in intervention time. In these patients, brachial or radial puncture sites are well established alternatives to the femoral one and could be helpful. Nevertheless, this finding could also be by chance, as catheterization of PA is only one section of the whole intervention and there are other important parts during PAE which influence radiation dose. If these angles really have such effect remains unclear.

Compared to other important centres and larger collectives, our technical parameters are equivalent^26,28^. Additionally, during our procedures, the need for additional CBCT in the common iliac artery on both sides could be reduced over time. Whereas in the first half of our patient collective CBCT from the internal iliac artery was performed by default for comparison, correlation, and 3D-roadmap, we could reduce the number of CBCT scans significantly in the second half. Information extracted from MRA were sufficient for identifying PA origin and CBCT from the iliac artery was redundant. CT was only necessary in the PA to exclude collateral vessels and to quantify embolization area. This leads to an additional reduction in radiation dose (that saves in mean 1440 ± 270 cGycm^2^ per CBCT in our collective)^29,30^. Intervention times between the first half and the second half of the included interventions differed significantly, probably explained by a learning curve. Thus, the combination of pre-interventional MRA and increasing experience may lead to further improvement of the intervention^31^.

When focusing on the different types of PA origin, results revealed no significant effect on procedural parameters. This stands in line with the results of previous published data^12–14^. There is a variety of different factors that influence the process and the difficulty of PAE. Time for probing of the internal iliac artery and its side branches and time for the embolization process itself are challenging parts of the intervention. Subsequently, technical, and procedural differences between the different PA types tend to be minor.

Some limitations of this study, besides the retrospective single centre design, need to be discussed. First, we did not report clinical outcome details for the patients. This paper focussed on technical aspects of the intervention more than on evaluating clinical success. Besides, we did not examine a control group without pre-interventional MRA to investigate on the direct advantage in terms of shorter intervention times due to an exact anatomic knowledge before performing PAE. As we focussed more on the predictive value of anatomic parameters measured in MRA, our project was not designed as a comparative study. We did not compare interventions with and without pre-interventional MRA, nor did we compare MRA with other imaging modalities such as CBCT or CTA. Further studies are needed to compare MRA with other imaging modalities. Next, Iliac tortuosity and elongation were measured subjectively as described above. Objective measurements of elongation in MRA could contribute to a better comparability, although to our knowledge, there is no established method to quantify this parameter in MRA. Also, atheroma or calcification severity could not be sufficiently assessed on MRI. Moreover, we did not investigate the impact of iliac artery stenosis on procedural parameters. As MRI often overestimates the severity of stenosis, we did not expect a significant effect and to our experience, most of the stenosis can be passed with 0.016’’-microwire and microcatheter. We observed a significant reduction in number of CBCT and radiation dose over the course of time. However, the division into two groups might seem arbitrarily and it is difficult to deduce if this reduction is explained by a learning curve or by the additional information of MRA or both. Nevertheless, improvement of technical skills, experience in image interpretation, and choice of material might lead to a reduction of radiation dose and intervention time.

We conclude that pre-interventional MRA provides useful and detailed anatomic information of pelvic arteries, which facilitates an adequate planning of PAE. Visual evaluation of vessel elongation was sensible to predict technical difficulty and could have an influence on the choice of materials (e.g., catheter type) and intervention time. However, this study was not able to show that MRA alone could predict embolization success. To exclude prostate cancer, mpMRI might be conducted in patients who are considered for PAE and have elevated PSA values anyway. In this setting, it seems practicable to acquire additional MRA for PAE planning.

### Supplementary Information

Supplementary Information.

## Data Availability

The datasets used and/or analyzed during the current study available from the corresponding author on reasonable request.

## Abbreviations

PAE: Prostatic artery embolization
BPH: Benign prostatic hyperplasia
MRA: Magnetic resonance angiography
DSA: Digital subtraction angiography
PA: Prostatic artery
DAP: Dose area product
CAK: Cumulative air kerma
CM: Contrast media
CBCT: Cone beam computed tomography
TURP: Transurethral resection of the prostate
ANOVA: Analysis of variance
CTA: Computed tomography angiography
mpMRI: Multiparametric magnetic resonance imaging
PIRADS: Prostate Imaging and Reporting Archiving Data System
PSA: Prostate specific antigen
T: Tesla
T1w: T1-weighted Imaging
T2w: T2-weighted Imaging
ADC: Apparent diffusion coefficient
TSE: Turbo spin echo
rs-EPI: Readout segmented echo-planar imaging
ss-EPI: Single slice echo-planar imaging
FLASH: Fast low angle shot
MIP: Maximum intensity projection
CFA: Common femoral artery
