# Supplementary material for: Value of magnetic resonance angiography before prostatic artery embolization for intervention planning
Source: Sci Rep. 2024 Apr 2;14:7758. doi: 10.1038/s41598-024-58207-3 (PMC10987590; doi:10.1038/s41598-024-58207-3)
Supplement: Supplementary file 1 — Supplementary Information. [file 41598_2024_58207_MOESM1_ESM.pdf]

Supp. Tab. 1: MRI sequences parameter.

Sequence	Localizer	T2w ax	T2w cor	T2w sag	Ptx-EPI DWI	T1w vibe iso ax
**TE (ms)**	106	102	99	101	67	6.47
**TR (ms)**	1000	3990	3880	4060	3500	2.46
**Slice thickness (mm)**	6	3	3	3	3	1.5
**Number of slices (n)**	24	32	22	20	24	208
**FOV (mm)**	380x380	160x160	170x170	170x170	91x150	300x300
**Flip angle (deg)**	20	150	137	134	90	10
**Acquisition time (min)**	0:43	5:19	4:16	3:23	3:35	4:06

Magnetom PRISMA, Siemens
ptx EPI = parallel transmit echo planar imaging; TE = time of echo; TR = time of repetition; FOV = field of view

Supp. table 2: Baseline characteristics.

	**Patients (n=78)**
**Age** years,<br>median (IQR)	71 (66 – 77)
**BMI** kg/m²,<br>median (IQR)	26.1 (23.8 – 28.7)
**PSA** ng/ml,<br>median (IQR)	5.5 (3.4 - 10.5)
**PIRADS classification**<br>Median (IQR)	2 (2 - 2)
**Volume** ml<br>Median (IQR)	88 (64 – 124)

BMI = Body mass index; PSA = prostate specific antigen; PIRADS = prostate imaging reporting and data system; IQR = Interquartile range

Supp. Tab. 3: PA type left versus right for DSA for patients with both left and right PA.

**DSA**		**right**					
		Type 1	Type 2	Type 3	Type 4	Other	Total
**left**	Type 1	14	3	2	4	0	23
	Type 2	2	11	0	0	0	13
	Type 3	3	4	10	1	0	18
	Type 4	6	4	1	7	0	18
	Other	2	0	0	0	0	2
	Total	27	22	13	12	0	74

DSA = digital subtraction angiography; PA = prostatic artery

Supp. Tab. 4: Cross table PA type DSA versus MRA.

		**DSA**					
		Type 1	Type 2	Type 3	Type 4	Other	Total
**MRA**	Type 1	38	0	3	1	1	43
	Type 2	5	33	0	2	0	40
	Type 3	3	1	28	1	0	33
	Type 4	4	0	0	26	0	30
	other	0	0	0	0	1	1
	total	50	34	31	30	2	147

Overall accuracy: (126/147, 86%; 95% CI: (0.79, 0.91)

DSA = digital subtraction angiography; MRA = magnetic resonance angiography

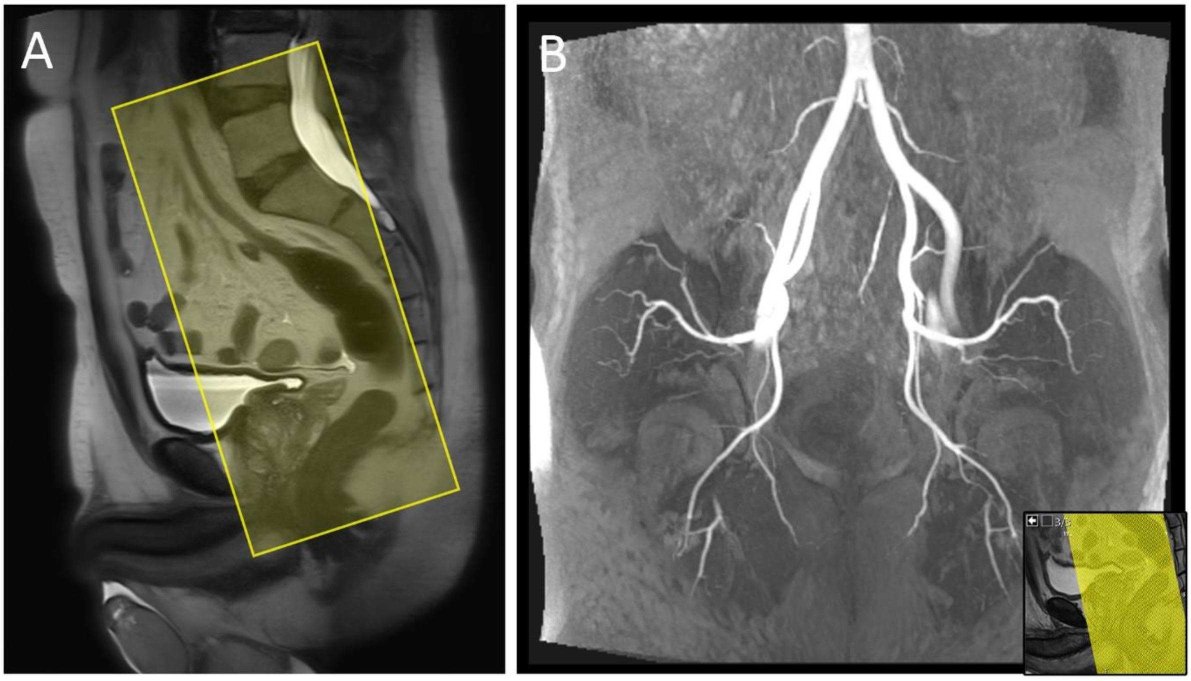

Supp. Fig. 1: Field of view (FOV) and Angulation of MRA sequence planning on a sagittal T2 image (A). 3D reconstruction of the pelvic MRA (B).
